# Current perspectives of lncRNAs in abiotic and biotic stress tolerance in plants

**DOI:** 10.3389/fpls.2023.1334620

**Published:** 2024-01-08

**Authors:** Xin Jin, Zemin Wang, Xuan Li, Qianyi Ai, Darren Chern Jan Wong, Feiyan Zhang, Jiangwei Yang, Ning Zhang, Huaijun Si

**Affiliations:** ^1^ State Key Laboratory of Aridland Crop Science, Gansu Agricultural University, Lanzhou, China; ^2^ College of Life Science and Technology, Gansu Agricultural University, Lanzhou, China; ^3^ Division of Ecology and Evolution, Research School Research of Biology, The Australian National University, Acton, ACT, Australia

**Keywords:** long noncoding RNA, small RNA, competing endogenous RNAs, epigenetic, abiotic stress, biotic stress

## Abstract

Abiotic/biotic stresses pose a major threat to agriculture and food security by impacting plant growth, productivity and quality. The discovery of extensive transcription of large RNA transcripts that do not code for proteins, termed long non-coding RNAs (lncRNAs) with sizes larger than 200 nucleotides in length, provides an important new perspective on the centrality of RNA in gene regulation. In plants, lncRNAs are widespread and fulfill multiple biological functions in stress response. In this paper, the research advances on the biological function of lncRNA in plant stress response were summarized, like as Natural Antisense Transcripts (NATs), Competing Endogenous RNAs (ceRNAs) and Chromatin Modification etc. And in plants, lncRNAs act as a key regulatory hub of several phytohormone pathways, integrating abscisic acid (ABA), jasmonate (JA), salicylic acid (SA) and redox signaling in response to many abiotic/biotic stresses. Moreover, conserved sequence motifs and structural motifs enriched within stress-responsive lncRNAs may also be responsible for the stress-responsive functions of lncRNAs, it will provide a new focus and strategy for lncRNA research. Taken together, we highlight the unique role of lncRNAs in integrating plant response to adverse environmental conditions with different aspects of plant growth and development. We envisage that an improved understanding of the mechanisms by which lncRNAs regulate plant stress response may further promote the development of unconventional approaches for breeding stress-resistant crops.

## Introduction

Ribonucleic acid (RNAs) are divided into coding RNAs and non-coding RNAs (ncRNAs). The ncRNAs constitute a class of RNA which includes microRNAs (miRNAs), small interfering RNAs (siRNAs), long non-coding RNAs (lncRNAs), and circular RNAs (circRNAs). The different types of aforementioned ncRNAs are involved in transcriptional and post-transcriptional regulation of gene expression, as well as regulation of RNA stability and translation (Wang et al., 2017; Qiu et al., 2019; Waititu et al., 2021). Instances where lncRNAs are transcribed by RNA polymerase II (Pol II) and/or Pol IV/V are also known (Rai et al., 2019). LncRNAs can be further classified as antisense lncRNAs or intron lncrnas (lincRNAs), depending on their genomic location (Wierzbicki et al., 2021).

LncRNA are heterogeneously regulated transcript that has emerged as a versatile regulator in a variety of development processes (Zhang et al., 2021a; Cao et al., 2022; Ren et al., 2022; Ye et al., 2022). In *Brassica rapa*, 47 *cis*-acting lncRNAs and 451 *trans*-acting lncRNAs were identified during pollen development and fertilization. These lncRNAs were notably co-expressed with their target genes (Huang et al., 2018). In tomato, the silencing of two lncRNAs (lncRNA1459 and lncRNA1840) delayed fruit ripening (Zhu et al., 2015). Moreover, Li et al. (2018) found that tomato fruit ripening was significantly inhibited with CRISPR/cas9-mediated mutation of lncRNA1459. In cucumber, lncRNA *CsM10* is specifically expressed in male cultivated cucumber species and may play a role in sexual differentiation (Cho et al., 2006). Beyond developmental processes, emerging findings indicate that lncRNAs also fulfill important roles in abiotic and biotic stress (Moison et al., 2021; Song et al., 2021; Ye et al., 2022). In many plants, numerous stress-related lncRNAs have been found by high throughput sequence analyzes (Wu et al., 2019; Gong et al., 2021; Li et al., 2022). Similar to protein-coding genes, lncRNAs display stress-responsive differential expression in addition to tissue- and developmental-specific expression patterns. Furthermore, small RNAs may also influence lncRNA expression in abiotic stress. In wheat, the lncRNAs –*TalnRNA9* and *TalnRNA12* – could be regulated by 24 nt siRNAs (Xin et al., 2011).

LncRNAs and miRNAs are both non-coding RNAs, and lncRNAs have the potential to act as competing endogenous RNAs (ceRNAs) to regulate the expression of target genes (Shuai et al., 2014). Additionally, lncRNAs can be targeted by miRNAs and may also serve as an endogenous target mimic (eTM) and/or possible miRNA precursors (Wu et al., 2013; Li et al., 2017). By analyzing competing relationships between mRNAs and lncRNAs, genome-scale lncRNA-mediated ceRNAs networks have been inferred in many plants (Fan et al., 2018; He et al., 2020; Ren et al., 2022). For instance, lncRNA regulates the expression of heat stress response genes (e.g. HEAT-SHOCK PROTEIN, *HSPs* and HEAT STRESS TF, *HSFs*) by competitively binding to bra-miR159a or bra-miR172a in Chinese cabbage (Wang et al., 2019a). Certain lncRNAs also function as natural antisense transcripts (NATs) that are transcribed in an orientation opposite to that of protein-coding genes. Examples include Arabidopsis *MAS* and potato *StFLORE* (Zhao et al., 2018; Ramirez et al., 2021). Some ncRNAs produced by PolIV are precursors of siRNAs (Movahedi et al. (2015) but may also participate in scaffolding RNA by modulating the chromatin framework. Examples include the Arabidopsis lncRNA *COOLAIR* (Csorba et al., 2014) and *APOLO* (Moison et al., 2021). Many stress-responsive lncRNAs have also been identified by screening the genome-wide binding peaks of ChIP-seq data (Di et al., 2014). For example, 107 stress-responsive lncRNAs were inferred from the binding signals of PIF4 and PIF5 TFs ChIP-seq data. Many conserved sequence and enriched structural motifs in different functional lncRNAs groups were also identified. They include a UUC motif responding to salt and a AU-rich stem-loop correlated with cold stress response (Di et al., 2014). As such, these conserved elements might also be responsible for the stress-responsive functions of lncRNAs.

In light of their emerging roles, lncRNA holds promise as a viable target for enhancing agronomic traits and stress tolerance in crop breeding endeavors. In this review, we systematically summarize the plant lncRNAs involved in stress responses and their crucial, but often overlooked, roles in plants.

## Abiotic stress-associated lncRNAs in plants

**Figure 1:**
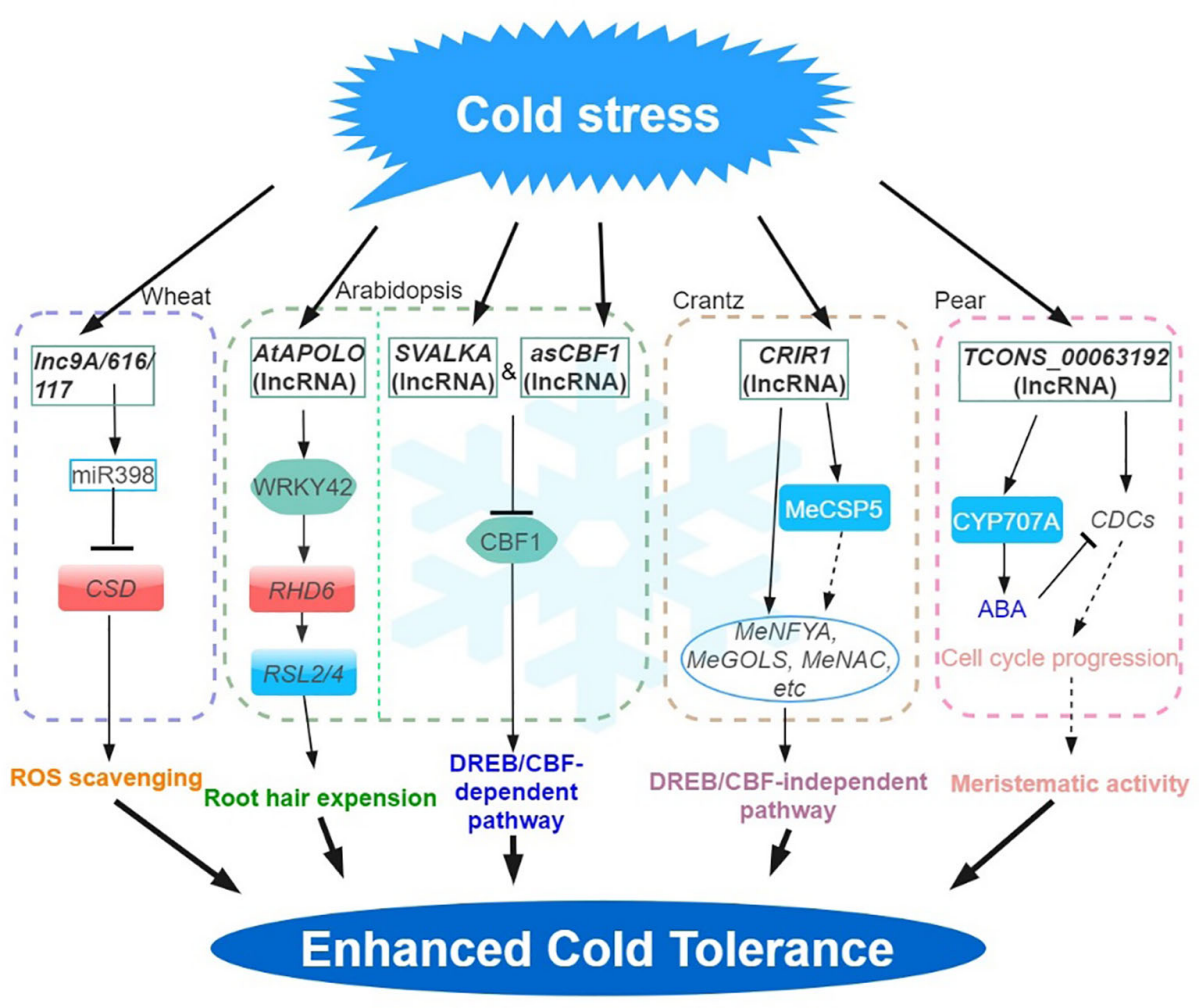
Regulatory network of the lncRNAs involved in cold stress. Cold stress-responsive lncRNAs in Arabidopsis, wheat, crantz and pear are shown in the chart. The arrows and hammer represent positive and negative regulation, respectively. APOLO, lncRNA AUXIN-REGULATED PROMOTER LOOP; MeGOLS, GALACTINOL SYNTHASES; DREB, DEHYDRATION-RESPONSIVE ELEMENT (DRE) BINDING FACTOR; CBF, C-REPEAT (CRT)-BINDING FACTOR; CSP, COLD SHOCK DOMAIN‐CONTAINING PROTEIN; ROS, REACTIVE OXYGEN SPECIES.

**Figure 2:**
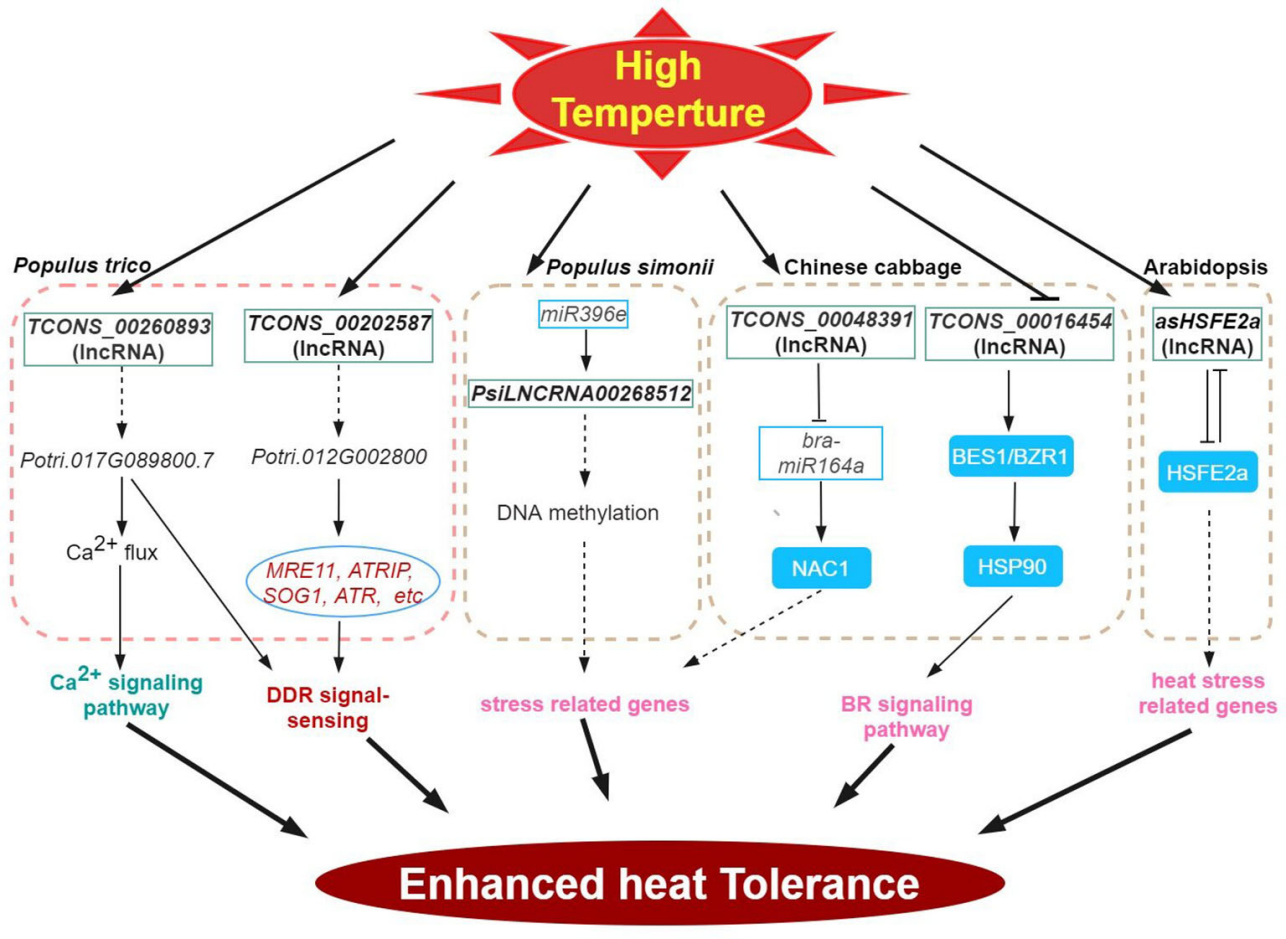
High-temperature stress signaling and responses of lncRNAs in plants. HSP, HEAT-SHOCK PROTEIN; BES1, BRASSINOSTEROID INSENSITIVE 1EMS-SUPRESSOR 1; BZR1, BRASSINAZOLE RESISTANT 1; HSF, HEAT STRESS TF.

**Figure 3:**
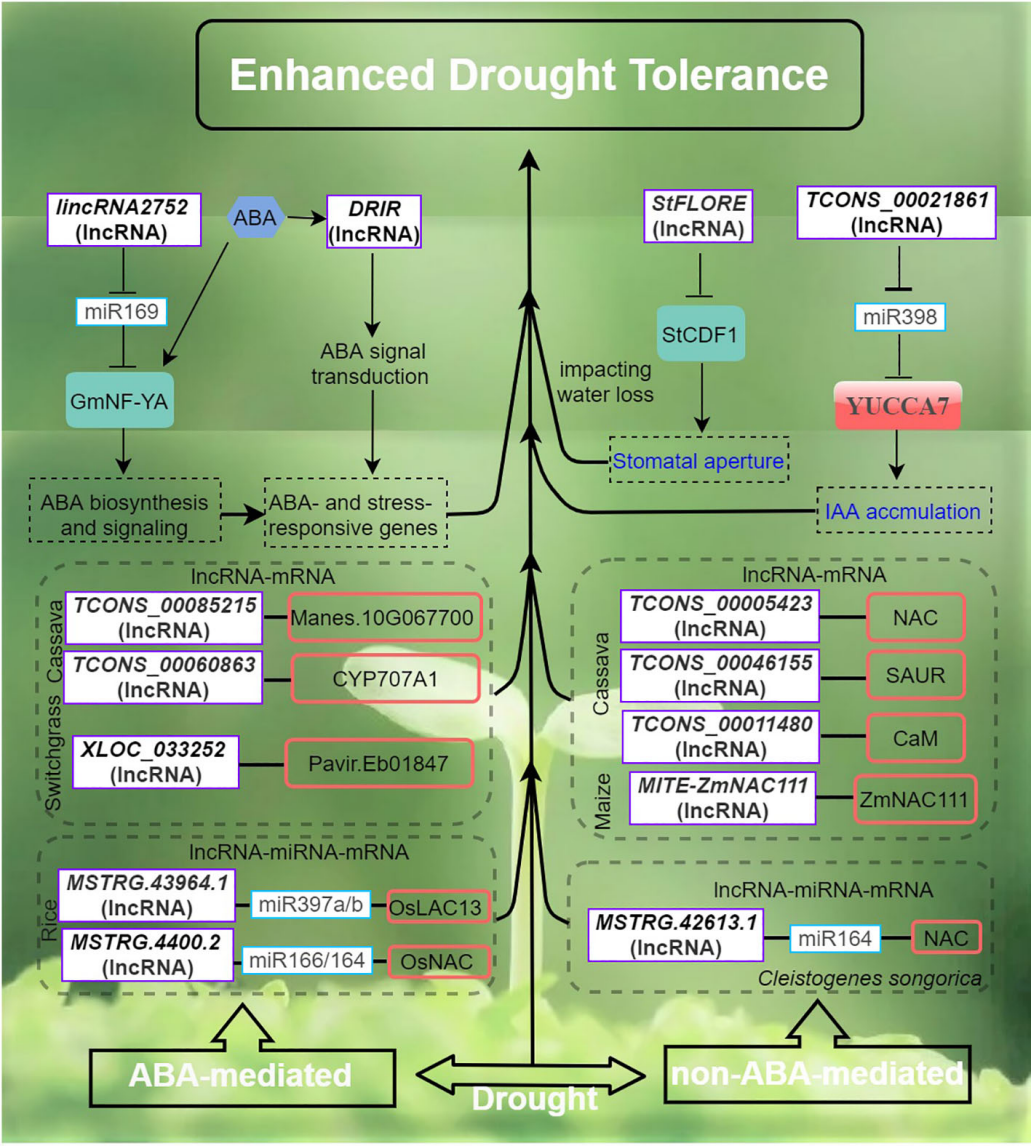
Drought stress signaling and responses of lncRNAs in plants are primarily by ABA-dependent and ABA-independent pathways. The arrows and hammer represent positive and negative regulation, respectively. ABA, abscisic acid; StCDF1, CYCLING DOF FACTOR 1; DRIR, DROUGHT INDUCED lncRNA; StFLORE, The StCDF1 locus also codes for an antisense lncRNA; YUCCA7, flavin monooxygenase gene.

**Figure 4:**
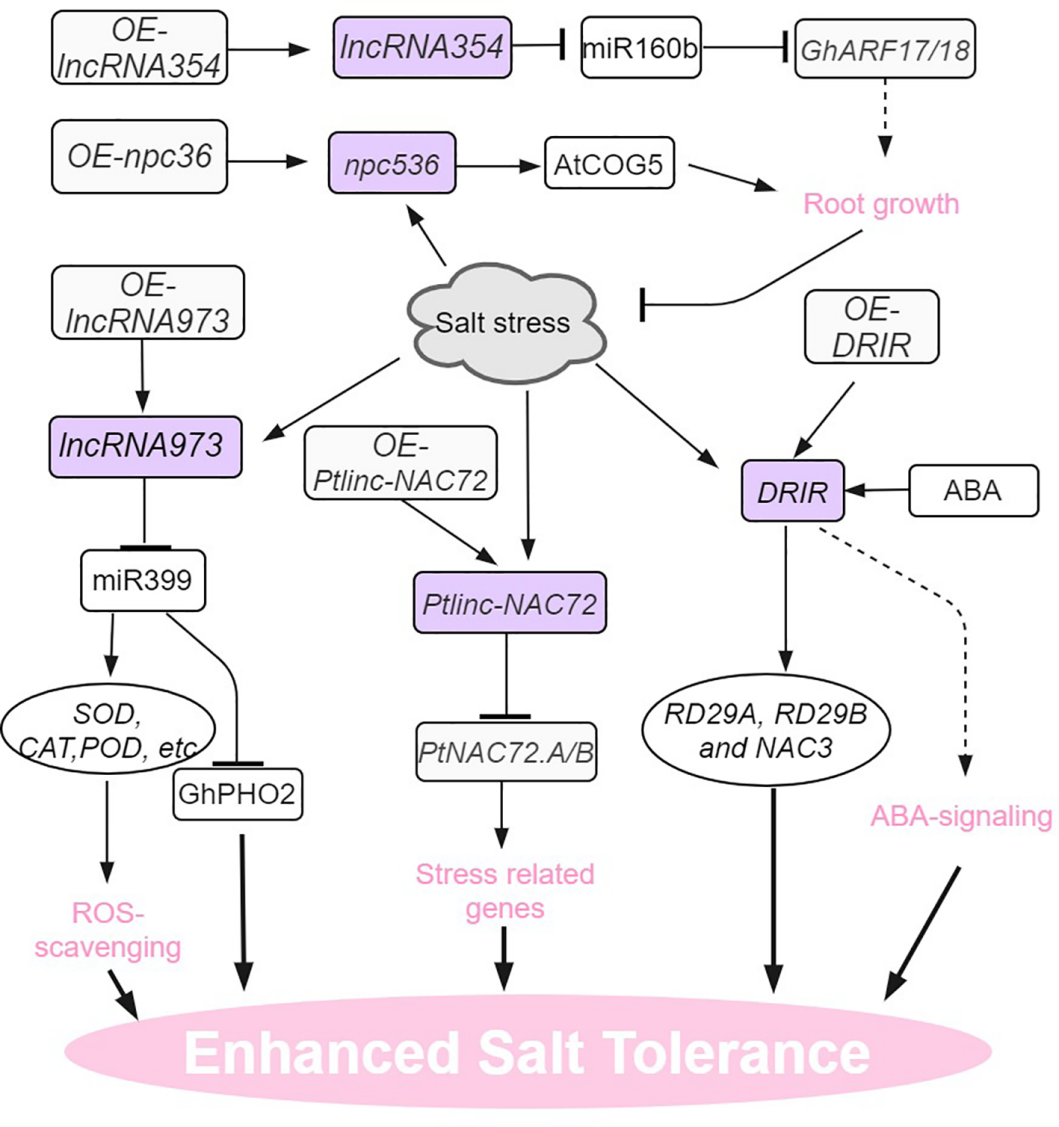
Salt stress signaling and responses of lncRNAs in plants. AtCOG5, CONSERVED OLIGOMERIC GOLGI COMPLEX 5; DRIR, DROUGHT INDUCED lncRNA; RD29, RESPONSIVE TO DESSICATION 29. The arrows and hammer represent positive and negative regulation, respectively.

Adverse environmental conditions, such as extreme temperature, drought, and high salinity, negatively impact plant growth and development. In response to abiotic stress, a myriad of stress-responsive proteins and regulatory factors are often highly induced (Zhu, 2016; Zhang et al., 2022a). Similarly, lncRNAs fulfilling roles in the regulation of abiotic stress responses share such characteristics (Wang et al., 2019a; Yang et al., 2022; Ye et al., 2022). The roles of many lncRNAs in the context of abiotic stress responses have now been well investigated. (**Figures 1**–**4**). This is in part enabled by the advent of next-generation sequencing technologies that have provided the identification of many lncRNAs from various plant species, growth and development processes (e.g. root development, flowering time control), and stress conditions (Zhao et al., 2018; Gong et al., 2021; Moison et al., 2021; Li et al., 2022). In these studies, we also found that some transcription factors are widely involved in the regulation of lncRNA in different stress responses, such as lncRNA-NAC in drought, salt stress, and temperature stress. the implication is that lncrnas participate in the common regulatory network in various abiotic stresses by regulating some key members of the network.

## LncRNAs response to extreme temperature stress in plants

### Low temperature

Low-temperature stress constitutes a prominent factor influencing plant growth and development, leading to a substantial reduction in crop production (Gong et al., 2020; Ding and Yang, 2022; Zhang et al., 2022a). In plants, C-REPEAT (CRT)-BINDING FACTOR/dehydra-RESPONSIVE ELEMENT (DRE) -BINDING FACTOR (CBF/DREB) -mediated transcriptional regulatory cascade pathway is the main low temperature signaling pathway. This mechanism is key to inducing a series of cold response (COR) genes (Shi et al., 2015). In Arabidopsis, *asCBF1* – a cryptic lncRNA (overlapping *CBF1* on the antisense strand) produced by RNAPII read-through transcription of the lncRNA, *SVALKA –* represses *CBF1* and promotes cold acclimation. This *SVALKA-asCBF1* cascade pathway provides a negative feedback for plants to adapt to low temperatures (by maximizing cold tolerance while reducing adaptation costs) through the strict control of *CBF1* expression and timing (Kindgren et al., 2018). In grapes, 487 and 326 lncRNAs were significantly up- and down-regulated during cold stress, respectively. Among them, 203 differentially expressed lncRNAs were predicted to regulate 326 target genes. Among them, many differentially expressed lncRNAs were predicted to target stress response-related genes such as *CBF* and *WRKY* transcription factors, late embryogenesis abundant (LEAs), and peroxisome biogenesis-related genes (Wang et al., 2019b).

In cassava, a total of 316 lncRNAs were characterized as cold‐related lncRNAs (Li et al., 2022). Functional analysis shows that the trans-acting lncRNA, CRIR1 can confer cold stress tolerance by interacting with a cold shock domain protein (MeCSP) to improve translation efficiency at low temperatures. Many cold‐related genes (e.g. *MeNAC*, *MeNF-YA* and *MeGOLS*, GALACTINOL SYNTHASES), were upregulated in WT and *CRIR1*-OE plants during cold stress, however, CBF‐dependent pathway cold stress‐related marker genes (e.g. *MeCBFs*, *MeCOR* and *MeICE1*) were not differentially expressed (DE). This suggested that *CRIR1* enhanced plant cold tolerance via a CBF‐independent pathway, likely via the auxin signaling (Li et al., 2022).

In *Arabidopsis*, pre-mRNA alternative splicing (AS) and lncRNA expression changes have been associated with stress response. For example, by exploiting a new Arabidopsis transcriptome prediction (named AtRTD2), regulatory and post-transcriptional regulation of lncRNA gene expression in response to cold stress has been possible (Calixto et al., 2019). Briefly, the analysis of a comprehensive RNA-seq time-series dataset identified 135 lncRNA genes with cold-dependent DE and/or differential AS of lncRNAs. By carrying out experiments on plants cultivated under different temperatures and employing high-resolution time-course analysis spanning the initial three hours before temperature reduction, it was demonstrated that the AS of specific lncRNAs exhibits a remarkable sensitivity to even minute temperature fluctuations. This finding suggested that the expression of these lncRNAs are subject to stringent regulation (Calixto et al., 2019). In addition, the identification of a cold-repressive lincRNA159 has been made possible using strand-specific RNA-seq. Functional analysis shows that lincRNA159 is the target mimics of miR164, and 16 of the 682 lncRNAs were identified to act as ceRNA in cassava (Li et al., 2017). *MiR398* is reported to respond to diverse abiotic stresses (Leng et al., 2017). In wheat, the ceRNAs –*lncR9A*, *lncR117*, and *lncR616* – interact with *miR398*, to regulate the expression of *COPPER/ZINC SUPEROXIDE DISMUTASE 1* (*CSD1*) and improve tolerance to cold stress (Lu et al., 2020).

Natural antisense transcripts (NATs) is another type of lncRNAs playing significant roles in various physiological processes. NAT lncRNAs can serve as scaffolds to facilitate chromatin conformation changes, DNA methylation, and histone modifications (Chen et al., 2012). The induction of *MAS*, a NAT lncRNA transcribed from the *MADS AFFECTING FLOWERING4 (MAF4)* locus, occurs as a result of cold and leads to the upregulation of *MAF4* expression at the transcriptional level. The interaction of MAS with WDR5a (a core component of the COMPASS-like complexes) further mediates the recruitment of WDR5a to *MAF4*, thus, enhancing H3K4me3 (Zhao et al., 2018). These findings demonstrate the involvement of NAT-lncRNAs in the regulation of gene expression during the vernalization response. Some plant lncRNAs also mediate the regulatory pathway of chromatin modification (lncR2Epi) (Wang et al., 2016). Previous studies showed that cold-induced lncRNA *COOLAIR* can inhibit the expression of *FLOWERING LOCUS C* (*FLC*) in *Arabidopsis* under cold stress through the lncR2Epi regulatory pathway (Heo et al., 2013; Csorba et al., 2014). During vernalization, *COOLAIR* and *COLD ASSISTED INTRONIC NONCODING RNA* (*COLDAIR*) are produced by the *FLC* gene. *COOLAIR* is a NAT lncRNAs of *FLC*, and the expression level increased rapidly in the early vernalization stage, effect on *FLC* transcriptional shutdown is independent histone H3 lysine 27 trimethylation (H3K27me3) accumulation, but mediates reduction in H3K36me3 at *FLC* during vernalization (Kim and Sung, 2012; Csorba et al., 2014). In contrast, *COLDAIR* is transcribed by the first intron of the *FLC* gene, is induced at a later stage of low temperature conditions and continuously increases after vernalization (Kim and Sung, 2012). In further analysis, *COLDWRAP* (*COLD OF WINTER-INDUCED NONCODING RNA FROM THE PROMOTER*), an *FLC* promoter-derived lncRNA, is induced by vernalization and functions with the *COLDAIR* (Kim and Sung, 2017). *COLDAIR* cooperates with *COLDWRAP* resulting in the repression of *FLC* gene by forming an intragenic chromatin loop, leading to the establishment of high H3K27me3 (Swiezewski et al., 2009; Kim and Sung, 2017). In Arabidopsis, the interaction of the lncRNA *AUXIN-REGULATED PROMOTER LOOP* (*APOLO*), with WRKY42 coordinates the activation of *RHD6* by binding to the *RHD6* promoter, which in turn modulates local chromatin 3D conformation, leading to root hair cell elongation in response to low temperatures (Moison et al., 2021). Furthermore, bud dormancy serves as a crucial and intricate protection mechanism employed by plants in winter, like grapes, apples and pears (Masocha et al., 2020; Wang et al., 2020; Li et al., 2021). In pear, the potential roles of lncRNAs in regulating the dormancy release by interactions with mRNA and miRNAs that are associated with the ABA degradation pathway have also been proposed following simulated chilling temperature regimes to induce buds to enter different dormancy states (Li et al., 2021).

### High temperature

Like low temperature, heat stress significantly impacts the growth and development of plants, thus, limiting global crop productivity (Gong et al., 2020; Ding and Yang, 2022; Lesk et al., 2022; Zhang et al., 2022a) At high temperatures, plants often induce genes encoding reactive-oxygen-scavenging (ROS) enzymes and the regulatory proteins (e.g. TFs and protein kinases), among others (general plant heat stress review here). Accumulating evidence has shown the importance of lncRNAs in plant responses to heat stress. Several heat stress response co-expression networks between lncRNA and mRNA have been inferred by analyzing cis-acting lncRNAs (Bhatia et al., 2020; He et al., 2020; Hu et al., 2022). Consistently, many TF genes were enriched in such networks providing support for the regulatory role of lncRNAs in the regulation of TFs during heat stress response potential targets of heat stress-responsive lncRNAs (204 transcripts) have also been inferred in poplar (Song et al., 2020). In wheat, Xin et al. (2011) characterized 77 lncRNAs during heat stress, and one candidate, *TahlnRNA27* was identified as a putative miRNA precursor. lncRNAs can be further classified into poly(A)+ and poly(A)- transcript type. Compared with poly(A)+ lncRNAs, the poly(A)- lncRNAs exhibit comparatively shorter transcripts and lower levels of expression and show notable specificity in their expression patterns in response to stresses (Di et al., 2014). In *Arabidopsis*, 245 DE poly(A)+ and 58 poly(A)- lncRNAs were responsive under various stress, however, of these, only 15 were heat-responsive (Di et al., 2014). In B. rapa, many of the 192 predicted target genes of the 34 DE lncRNAs identified under heat stress were also heat responsive (Song et al., 2016a). Comparative phenotype and transcriptome analysis of the heat-resistant elite maize inbred line under heat stress revealed 993 heat-responsive DE lncRNAs with close to a thousand predicted cis-regulated target genes that share strong co-expression (Hu et al., 2022). Many important biological processes and pathways involved in heat response including stress response, hormone signaling, metabolism, photosynthesis and spliceosome were also highly enriched (Hu et al., 2022).

DNA methylation plays an important role in various plant stress responses. In an abiotic stress-tolerant poplar (*Populus simonii*), comparative methylome and gene expression analysis under four abiotic stresses (cold, osmotic, heat, and salt stress) enabled the prioritization of stress-specific differentially methylated regions (SDMRs) containing 17 lncRNA genes. In particular, the epigenetic pathway-mediated regulation of SDMR162 region encompassed the *miR396e* gene and *PsiLNCRNA00268512*. Stable predicted interaction energies between *PsiLNCRNA00268512* and *miR396e* indicate that *PsiLNCRNA00268512* may serve as a target mimic regulating *miR396e* levels (Song et al., 2016b). In Chinese cabbage, heat-responsive lncRNA (*TCONS_00048391*) regulates the expression of *bra-miR164a* by acting as an endogenous target mimic (eTM) of *bra-miR164a*. Furthermore, this lncRNA is predicted to function as a sponge for miRNA binding or as a ceRNA that targets *NAC1* (Wang et al., 2019a).

The HEAT STRESS TF (HSFs) is the central regulator of heat stress response (Scharf et al., 2012; Zhu, 2016; Chen et al., 2018). For example, Arabidopsis *HSFB2a* is expressed in the female gametophyte and regulates vegetative and gametophyte development processes (Wunderlich et al., 2014). Transgenic experiments revealed that the NAT lncRNA of *HSFB2a*, *asHSFB2a* specifically counteracts *HSFB2a* expression when overexpressed. And showed improved biomass production. Conversely, overexpression of *HSFB2a* impaired biomass production. Following heat stress, the repressive effects of *HSFB2a* is counteracted by the antisense regulation of *HSFB2a* thereby restoring growth and further development (Wunderlich et al., 2014). In another example, the HSP90 participates in the regulation of BR signaling and BR responsive genes through the interaction with BRASSINOSTEROID INSENSITIVE 1EMS-SUPRESSOR 1 (BES1)/BRASSINAZOLE RESISTANT 1 (BZR1) TF, (Shigeta et al., 2015). In the Chinese cabbage, the lncRNA *TCONS_00016454* may be involved in conferring heat tolerance via BR signaling as this lncRNA showed antagonistic expression patterns with its target, a *BES1/BZR1* homolog in response to high temperatures in this system (Wang et al., 2019a).

In plants, intracellular concentration of free Ca^2+^ exhibits fast and stimulus-specific patterns (Zhang et al., 2022a). In *Populus simonii*, the lncRNA *TCONS_00260893* is involved in the regulation of Ca^2+^ flux and Ca^2+^ signaling under stress conditions by interfering with the target gene transcription encoding a cyclic nucleotide-regulated ion channel family protein (Potri.017G089800) via RNA interference and/or as RNA scaffolds. Heterologous expression of Potri.017G089800 in Arabidopsis enhanced photosynthetic protection and recovery, inhibited membrane peroxidation, and suppressed DNA damage in response to heat stress (Song et al., 2020). Potential ceRNA functions of lncRNAs and circRNAs in response to high-temperature stress have also been presented for cucumber (He et al., 2020).

## LncRNAs response to drought stress in plants

Drought poses a significant global concern in agriculture as it typically leads to reduced crop yield and quality (Gupta et al., 2020; Zhang et al., 2022a). LncRNAs are believed to play crucial roles in the regulation of gene expression in plants exposed to drought stress. To date, various lncRNAs regulatory pathways of plant drought tolerance and the functions of plant lncRNAs and their ceRNA pairs in response to drought have been elucidated (Shuai et al., 2014; Qin and Xiong, 2019). In rice, strand-specific sequencing and small RNA sequencing have enabled the identification of putative lncRNA, miRNA and mRNA associated with drought resistance and the construction of an integrated ceRNA network (Yang et al., 2022). A total of 191 lncRNAs and 32 miRNAs were found to be DE in drought-stressed rice. Interestingly, up to 16 drought-specific lncRNAs were predicted to target one drought-responsive gene, Os05g0586700 potentially implicated in plant membrane repair during drought (Yang et al., 2022). In another study, a rice drought-responsive ceRNA network, consisting of 40 lncRNAs, 23 miRNAs, and 103 mRNAs, was also presented (Chen et al., 2021). Particularly, lncRNA TCONS_00021861 could regulate YUCCA7 by sponging miR528-3p, thereby validating its potential role as ceRNA. This interaction subsequently triggers the activation of the IAA biosynthetic pathway, leading to the acquisition of drought stress tolerance.

In peanuts, integrated ceRNA networks have also been established with comparative transcriptomics between varieties with divergent levels of drought tolerance (Ren et al., 2022). A total of 673 ceRNA pairs were inferred in the drought-tolerant variety, and the resulting ceRNA network analysis identified six lncRNAs, namely *MSTRG.70535.2*, *MSTRG.86570.2*, *MSTRG.86570.1*, *MSTRG.100618.1*, *MSTRG.81214.2*, and *MSTRG.30931.1*, which were recognized as central nodes in the network. Components of this specific ceRNA network may have significant implications in enhancing the drought tolerance of peanuts (Ren et al., 2022). Similarly, comparative transcriptome analysis of two Tibetan wild barley with contrasting drought tolerance revealed 535 DE lncRNAs with distinct expression profiles under drought stress. A total 503 and 776 potential lncRNA-mRNA pairs in *cis*- and *trans*-acting mode, respectively were confidently inferred. Significant enrichment in molecular functions such as plant hormone signal transduction, kinase signaling, and ascorbate/aldarate metabolism characterized the majority of predicted target genes. One serine/threonine-protein kinase SMG1 in particular was predicted to be targeted by multiple lncRNA and thus hypothesized to undepin the drought-tolerant cultivar (Qiu et al., 2019). Similarly, in rapeseed, a comparative transcriptomic analysis of lncRNA changes between drought-tolerant and drought-sensitive cultivars responding to water deficit stress has also been performed (Tan et al., 2020). LncRNA-mRNA interaction network analysis identified 34 TFs corresponding to 126 DE lncRNAs in the drought-tolerant cultivar, and 45 TFs corresponding to 359 DE lncRNAs in the drought-sensitive ones These increased understanding of plant lncRNAs in response to drought stress holds significant value for advancing the investigation of lncRNA functionality and underlying processes in the context of drought stress.

The activation of stress-responsive genes is facilitated by both ABA and dehydration-induced primary and secondary signals, which operate through a complex network of ABA-dependent and ABA-independent signaling pathways (**Figure 3**). This network encompasses various processes, including sensing, signal transduction, and gene activation (Xiong et al., 2002; Zhu, 2016). In Arabidopsis, *DROUGHT INDUCED lncRNA* (*DRIR*), is specifically and strongly induced by drought, salt stress and ABA treatment, while remaining lowly expressed level under non-stress conditions (Qin et al., 2017). The T-DNA insertion mutant *drir^D^* and transgenic *DRIR* overexpressing lines in Arabidopsis showed that *DRIR* exerts a positive role by restricting transpirational water loss and enhancing drought tolerance. Furthermore, the *drir^D^* mutant and the OE-seedlings exhibited heightened sensitivity to ABA compared to the wild type (WT). Transcriptome analysis revealed significant modulation of genes associated with ABA signaling, water transport, and other stress-relief processes in both the *drir^D^* and the DRIR overexpressing lines (Qin et al., 2017).

The potato CYCLING DOF FACTOR 1, *StCDF1* in conjunction with its lncRNA counterpart, *StFLORE* (StCDF1 locus encodes for an antisense lncRNA), plays a role in the regulation of water loss by influencing stomatal development and diurnal opening (Ramirez et al., 2021). Both naturally occurring and CRISPR-Cas9 mutations in the *StFLORE* transcript result in plants that exhibit heightened sensitivity to water-limiting situations. Conversely, a higher level of *StFLORE* expression achieved either through the overexpression of *StFLORE* or the downregulation of *StCDF1*, leads to enhanced drought tolerance by mitigating water loss. Taken together, the *StCDF1–StFLORE* locus plays a significant role in both vegetative reproduction and water homeostasis (Ramirez et al., 2021). In *Populus trichocarpa*, drought-responsive *lincRNA2752* could reduce the level of *ptc-miR169* by acting as a target mimic of *ptc-miR169* (Shuai et al., 2014). *MiR169* is known to play an important role in drought stress by regulating TF NF-YA in plants (Ni et al., 2013). Therefore, the regulation network of *lincrna2752* on drought resistance may involve both *miR169-* and NF-YA-associated mechanisms. In maize, tissue- and developmental-stage spatio-temporal transcriptional dynamics of drought stress-responsive lncRNAs have also been investigated (Pang et al., 2019). Interestingly, the developmental stage showing the most drought-responsive lncRNA DE involves the transition from the vegetative stage to the reproductive stage. New insights into lncRNA-mediated gene regulation in drought response in this system were also gained. Particularly, one lncRNA-mRNA pair involving - *vpp4* (encoding a vacuolar (H^+^)-pumping ATPase subunit homolog) and its adjacent lncRNA *MSTRG.6838.1* sharing strong expression correlation was prioritized as one of many mechanisms underpinning the drought response (Pang et al., 2019).

## LncRNAs response to salt stress in plants

Salt stress is also a prominent environmental factor that has a significant impact on the growth and development of plants (Zhu, 2016; Gong et al., 2020; Zhang et al., 2022a). In *Populus trichocarpa*, multi-tissue (leaves, stems and roots) RNA-seq enabled the identification of 1183 salt-induced DE lncRNAs (Ye et al. (2022). One lncRNA in particular, *Ptlinc-NAC72* plays a negative role in the salt tolerance of this species. When exposed to long-term salt stress, *Ptlinc-NAC72* exhibited the capacity to cis- and trans-regulate salt-responsive genes. For example, *Ptlinc-NAC72* can directly upregulate *PtNAC72.A/B* expression by recognizing the GAAAAA tandem elements in the 5’-UTR of *PtNAC72.A/B*. Induction of *PtNAC72.A/B* in turn confers resilience of the plant to salt stress (Ye et al., 2022). In arabidopsis, *npc536* was identified as a salt-responsive lncRNA, which is observed to be upregulated in both roots and leaves when subjected to salt treatment. Furthermore, the overexpression of *npc536* has been found to promote root growth under conditions of salt stress, presumably through its involvement in regulating the translation of *CONSERVED OLIGOMERIC GOLGI COMPLEX 5* (*AtCOG5*) mRNA (Ben Amor et al., 2009).

In upland cotton, endogenous target mimic (eTM) analysis indicates that *LncRNA354* had a potential binding site for *miR160b* (Zhang et al., 2021a). Overexpression of lncRNA354 in transgenic Arabidopsis plants reduced tolerance to salt stress by affecting the expression of *miR160b* and target auxin responsive factors (*ARFs*). The transgenic plants overexpressing *GhARF17/18* target genes also shared this salt-sensitive phenotype. On the contrary, silencing *lncRNA354* and targets *GhARF17/18* showed taller plants and enhanced the tolerance to salt stress (Zhang et al., 2021a). In addition to its role in drought tolerance, Arabidopsis lncRNA *DRIR* is also salt-stress inducible and confers salinity stress tolerance (Qin et al., 2017). In salt-treated *drir^D^* and DRIR overexpressing seedlings, the expression of stress-related genes like *P5CS1*, *RD29A*, *RD29B*,and *NAC3* increased dramatically. Unlike drought conditions, no ABA signaling pathway genes (like *ABI5*) were found indicating that *DRIR* regulates stress-related genes through different mechanisms during specific stress conditions (Qin et al., 2017). In cotton, salt-stress inducible *lncRNA973* increased salinity stress tolerance by regulating the expression of *miR399* and its target gene *GhPHO2*. Knocking out *lncRNA973* in cotton seedling roots significantly increased miR399 expression (Zhang et al., 2019).

## LncRNAs response to biotic stress in plants

The involvement of lncRNAs in various abiotic stress responses has been well-documented, while their contributions to biotic stress response are gradually emerging. In wheat, Xin et al. (2011) identified 125 lncRNAs during powdery mildew infection and heat stress. Among them, 48 lncRNAs showed responsiveness only to powdery mildew infection (designated TapmlnRNA.) while 23 lncRNAs demonstrated responsiveness to both powdery mildew infection and heat stress (designated TalnRNA). Four lncRNAs (*TalnRNA5*, *TapmlnRNA8*, *TapmlnRNA19*, and *TahlnRNA27*) were shown to form stable hairpin structures and were identified as putative precursors of miRNA. Among them, *TapmlnRNA8* and *TapmlnRNA19* showed specificity toward powdery mildew infection (Xin et al., 2011). More recently, disease-responsive (e.g. to Rice Black-Streaked Dwarf Virus and Rice Stripe Virus) lncRNA profile was investigated in rice (Cao et al., 2022). A total of 21 lncRNAs were found to be up-regulated in response to both virus and putative targets including an excess of 1,000 co-regulated transcripts involved in transcriptional regulation, plant hormone signal transduction, phenylpropanoid biosynthesis, and plant-pathogen interaction terms (Cao et al., 2022).

In tomato plant, predicted ceRNA networks involving *Phytophthora infestans* resistance have been presented (Cui et al., 2020). One lncRNA in particular, *LncRNA40787* was strongly up-regulated upon infection with *P. infestans*. Additionally, *LncRNA40787* was predicted as a ceRNAs of *miR394* as it contains the endogenous target mimics (eTM) structure of *miR394*. Follow-up studies revealed miR394 plays a negative role in tomato resistance against *P. infestans* whereas lncRNA40787 positively regulates tomato defense response by acting as an eTM for miR394 to regulate *Leaf Curling Responsiveness* (*LCR*) by activating genes involved in JA biosynthesis components (Zhang et al., 2021b). A ceRNA network consisting of 5 lncRNAs, 5 miRNAs, 4 circRNAs, and 15 mRNAs has also been construcuted to provide the candidate ceRNAs in *Paulownia* and an in-depth understanding of the molecular mechanism responsible for Paulownia witches’ broom disease progression (Fan et al., 2018).

Recently, a pathogen infection-responsive lncRNA, termed salicylic acid biogenesis controller 1 (*SABC1*), was discovered to fine-tune salicylic acid biosynthesis, which in turn balances plant immunity and growth (Liu et al., 2022). CURLY LEAF (CLF), an essential component of polycomb repressive complex 2 (PRC2) responsible for catalyzing H3K27me3, was identified in *SABC1* RNA immunoprecipitation. Further molecular mechanism analysis indicates *SABC1* associates with the CLF protein and assists the recruitment of CLF-PRC2 to its neighboring NAC TF gene *NAC3* locus, thereby decreasing *NAC3* transcription via H3K27me3. In healthy plants, *SABC1* exerts a suppressive effect on SA production and plant immunity by downregulating the expression of *NAC3* and a key enzyme catalyzing salicylic acid biosynthesis, *isochorismate synthase 1*, however, upon pathogen infection, *SABC1* is downregulated and relieves the derepression of SABC1-mediated plant immune response (Liu et al., 2022). In cotton, Zhang et al. (2022b) leveraged co-expression network analysis to identify two herbivory-responsive lncRNA hub genes, lncA07 and lncD09, predicted to play a regulatory role in defense against aphid infestation. Functional analysis of these two lncRNA by CRISPR/Cas9 revealed that the phytohormone jasmonic acid was significantly decreased in CRISPR/Cas9 knock-out mutant of *lncD09* and *lncA07*. Furthermore, three candidate genes (*Ghir_A01G022270*, *Ghir_D04G014430*, and *Ghir_A01G022270*) were implicated in the regulation of the JA-mediated signaling pathway, which in turn underpins susceptibility to insect infestation (Zhang et al., 2022b).

## Conclusions and perspectives

The advent of next-generation sequencing technologies and complex bioinformatics analysis have enabled the identification of diverse lncRNAs and their predicted target genes in model plants to emerging crops but also those involved in various abiotic and biotic stress. Coupled with the confirmation of predicted lncRNA function in various plant systems using various reverse genetics approaches exceptional insights for the study of the complex lncRNA-mediated regulatory network controlling plant response tolerance have been gained. Here, we have highlighted a significant body of literature demonstrating the key role of lncRNAs in regulating various aspects of plant stress (abiotic and biotic stress), including stress perception, signal transduction, and downstream responsive pathways. These lncRNAs play multiple roles in plant stress through the different mechanistic strategies (as enhancers, decoys, scaffolds, and/or guides) that are tightly integrated into various plant stress response networks, including the regulation of transcription machinery and chromatin modification in promoter regions, modulation of TF-binding affinity in enhancer regions, RNA–DNA hybridization and modulation of chromatin topological structures, among others. Moreover, many conserved sequence motifs and structural motifs were found to be enriched in stress-responsive lncRNAs, suggesting their potential involvement in the stress-responsive functions of lncRNAs, however, detailed molecular mechanisms of stress regulation by these plant lncRNAs are very limited. Presently, validation of lncRNA function primarily relies on overexpression techniques, nonetheless, CRISPR/Cas holds great potential as an efficient and flexible method for investigating the function of lncRNAs (e.g., by creating functional loss- and/or gain-of-function mutants). It is anticipated that future investigations on lncRNA will continue to contribute valuable knowledge regarding plant stress along with viable strategies for the improvement of crop stress tolerance. With the ultimate goal to ensure global food security for future generations, it will be exciting to know whether lncRNAs hold the key to achieving a harmonious equilibrium between conferring resistance (i.e. an adequate level of stress response) and ensuring crop development, yield, and quality are not compromised.

## Author contributions

XJ: Writing – original draft, Writing – review & editing. ZW: Writing – original draft, Writing – review & editing. XL: Writing – original draft. QA: Writing – original draft. DW: Writing – review & editing. FZ: Writing – review & editing. JY: Writing – review & editing. NZ: Writing – review & editing. HS: Writing – review & editing.
